# Conducting Copper(I/II)-Metallopolymer for the Electrocatalytic Oxygen Reduction Reaction (ORR) with High Kinetic Current Density

**DOI:** 10.3390/polym10091002

**Published:** 2018-09-07

**Authors:** Sait Elmas, Wesley Beelders, Xun Pan, Thomas Nann

**Affiliations:** 1 Institute for Nanoscale Science & Technology, Flinders University, Bedford Park SA 5042, Australia; sait.elmas@flinders.edu.au (S.E.); caroline.pan@flinders.edu.au (X.P.); 2 Future Industries Institute, University of South Australia, Mawson Lakes SA 5095, Australia; wesley.beelders@unisa.edu.au; 3 School of Chemical and Physical Sciences, Victoria University of Wellington, Wellington 6420, New Zealand

**Keywords:** oxygen reduction reaction, ORR, electrocatalyst, copper, metallopolymer, hydroquinone, kinetic current density, fuel cells

## Abstract

The oxygen reduction reaction (ORR) is still the most research-intensive aspect of a fuel cell. The sluggish kinetics of the electrocatalysts toward the ORR requires large amounts of platinum to be used as cathode material, which calls for alternatives to replace or minimize the amount of the noble metals used. This study describes the synthesis and complete characterization of a copper metallopolymer (Cu MP) based on a conducting polymer (CP) and single-site catalytic centers for the electrocatalytic ORR. The copper (II) catalyst, embedded in a redox-active and conducting polymeric environment, was pursued as a potential candidate to replace noble metals in fuel cell applications. Performance studies at a rotating disk electrode (RDE) showed that the metallopolymer exhibited a direct four-electron reduction at potentials between −150 and −350 mV vs. the reversible hydrogen electrode (RHE) and high kinetic current densities of over 22.62 mA/cm^2^. The kinetic current densities obtained at the Cu MP electrode outperformed most of the reported state-of-the art electrocatalysts toward the ORR. Further analysis of the Cu/CP hybrid revealed the copper being largely reduced to the oxidation state +I.

## 1. Introduction

The growing energy demand and consumption worldwide still rely on fossil-based fuels. However, the depletive reserves in natural resources and the man-made greenhouse effect are calling for clean and sustainable energy conversion/storage technologies, for example, batteries [1,2,3], supercapacitors [4,5,6,7], solar power [8,9], and fuel cells [10,11]. The latter operate with high efficiency and low emission of pollutants [12]. In the Apollo missions in the 1960s, platinum (Pt)-based fuel cells were already being used as power source with great success. Since then, a large amount of work has been directed toward replacing the expensive Pt-based catalysts with cheaper, non-precious metal ones [13,14,15,16]. In a typical H_2_/O_2_ proton exchange membrane (PEM) fuel cell, hydrogen and oxygen react electrochemically at the anode and cathode, releasing electrical energy, heat, and water. In a typical configuration, both electrodes contain a Pt-based electrocatalyst to enhance the rate of the respective reaction [17]. Ideally, Pt should be replaced or minimized at both electrodes. However, since the oxygen reduction reaction (ORR) occurring at the cathode is much slower compared with the oxidation reaction, it requires a larger amount of platinum. By replacing or minimizing the cathode catalyst, fuel cells would become economically competitive, which could open up many new applications [12,13,18].

Several approaches to replace or minimize the amount of platinum in the catalyst have been taken, for example, the use of Pt-based alloys [19,20,21,22], transition metal chalcogenides [23,24], carbon (nanotube)-supported metal particles [25,26,27,28], metal-free carbon materials [15,29,30,31], enzymatic electrocatalytic systems [32,33,34], and conducting polymers such as poly(3,4-ethylenedioxy thiophene) (PEDOT), polyaniline (PAni), polypyrrole (PPy), and polythiophene (PTh) [35,36,37]. Some of the most promising non-platinum group metal (PGM) electrocatalysts are transition metal–nitrogen–carbon (M–N–C) materials (M = Co, Fe, Ni, Mn, etc.) [38,39,40,41]. Along with being inexpensive, they display promising ORR catalytic activity and reasonable stability and selectivity. However, one of the major drawbacks of such rigid electrocatalysts is leaching. Leaching occurs during startup/shutdown phases where the membrane shrinks and swells causing leaching of the catalytic layers of the fuel cell devices, thus decreasing the lifetime of the device dramatically [42]. Other significant losses in the catalysts’ performance during the long-term operation of rigid fuel catalysts has been attributed to the migration of Pt particles and the corrosion of the membrane, which is sandwiched between the cathode and anode in the membrane electrode assembly (MEA) [43,44]. Thus, alternative catalyst systems, where the catalyst is hosted in a flexible and conductive coordination environment, appeared to the authors as the logical step toward fuel cell research [45,46,47].

In this work, the authors developed a new class of ORR catalysts inspired by the work of Heeger et al. [48]. The catalyst systems comprised single-site active centers in a flexible, conducting polymer backbone, as opposed to rigid metal alloys or metal composites and agglomerates (pyrolized metals). By “doping” conducting polymers with single-site metal centers, the electrical conductivity was enhanced as well as the catalytic activity of various reactions.

Among the conductive polymers, the poly(heteroarylene) methines (PHAMs) are attractive candidates for hosting transition metals adjacent to the conducting polymer backbone. Furthermore, their ease of synthesis and structural modification makes them attractive to tailor hybrid organic-inorganic materials for electrocatalysis. Kingsborough and Swager introduced a salen-3,4-ethylenoxythiophene copolymer containing Co^2+/3+^ redox centers for the electrocatalytic ORR as a prime example of such an application, but they reported a sensitivity of the imine functionalities in the salen units to hydrolysis that undermined the high catalytic performance of the cobalt (II/III) metallopolymer. Degradation effects of the cobalt-CP electrocatalyst during extended times of electrocatalytic cycling were observed [45,49].

**Scheme 1:**
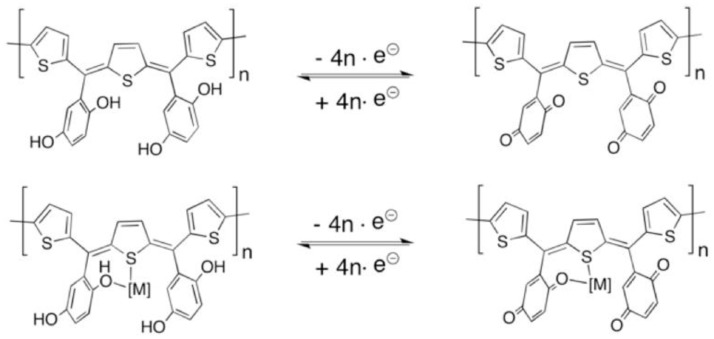
Reversible redox behavior of the poly-(thiophenyl)poly-hydroquinone (PThPHQ) and its metallopolymer [(PThPHQ)Cu], Cu MP. Each unit potentially provides four electrons for the reduction and accepts four electrons for the oxidation.

In this report, the authors describe the synthesis, characterization, and application of a novel copper-doped polymer, based on the PHAM structure. In addition to published PHAM structures, they introduced an electro-active quinone/hydroquinone moiety into the heteroarylene methine backbone [50,51,52], which provided a reversible two-electron redox response in a small potential window, thus expecting a four-electron redox response per repetition unit according to Scheme 1 [53,54,55].

In the presence of copper (II) as catalytic centers and oxygen as substrate, the concurrent redox behaviors of the intrinsically electron-conducting polymer and hybrid organic-inorganic material were compared. Surprisingly, the oxido-pincer-type [56,57] polymer–metal hybrid turned out to show high catalytic activity toward the oxygen reduction reaction with high kinetic current densities, which the authors believe to be a potential candidate to replace the current, expensive Pt-based catalysts.

## 2. Materials and Methods

### 2.1. Materials

1,4-dioxane (Sigma-Adrich, Castle Hill, Australia, 99.5%), thiophene (Sigma-Aldrich, >99%), 2,5-dihydroxy-benzaldehyde/gentisaldehyde (Sigma-Adrich >99%), sulfuric acid (Scharlau, Australia, 95%–97%), and Copper (II) bis-(tetrafluoroborate) dihydrate were used without further purification as obtained from suppliers.

NMR spectra were recorded on a Bruker Avance II 300 MHz spectrometer (Bruker, Karlsruhe, Germany) by using a triple-resonance 1H, nBB inverse probe head. All chemical shifts are quoted relative to tetramethyl silane (TMS).

UV/Vis spectra of the polymer and the copper metallopolymer were recorded on a Varian UV/Vis spectrometer (Varian, Sydney, Australia) in acetonitrile using a quartz cuvette with an optical path length of 1.0 cm.

Scanning electron microscopy (SEM) images were taken on a QEMSCAN system with a Zeiss Evo SEM platform combined with two silicon drift X-ray spectrometers (Bruker) and iDiscover v.5.2 software (FEI).

X-ray photoelectron spectroscopy (XPS) was performed with a Kratos Ultra DLD spectrometer (Kratos), using monochromatic Al kα radiation (*h*_ν_ = 1486.7 eV). The system was equipped with a magnetically confined charge compensation system (low energy electrons were confined and transported to the sample surface by magnetic field). Spectra were recorded using an acceleration voltage of 15 keV at a power of 225 W. Survey spectra were collected with a pass energy of 160 eV and an analysis area of 300 × 700 µm^2^. Data analysis was performed with CasaXPS software (Casa Software Ltd., Teignmouth, UK) and selected graphs were plotted with the Qti Plot software (Qti Plot ,Bucuresti, Romania).

Preliminary oxygen reduction reactions were recorded on an AUTOLAB potentiostat (Metrohm, Switzerland) using potentiostatic cyclic voltammetry methods. In this study, a glassy carbon (GC, 0.167 cm^2^) electrode acted as the working electrode (WE), a platinum rod as the counter electrode (CE), and Ag|AgCl (3 M KCl) as the reference electrode (RE). The cyclic voltammograms (CV) were recorded at 100 mV/s in the potential range of 0.2 to −1 V vs. Ag|AgCl (3 M KCl). The kinetics of the oxygen reduction reactions (ORR) was conducted on a rotating disk electrode (RDE) on a Solartron Electrochemical Interface SI 1287 potentiostat using a Rotating Electrode Speed Control (PINE Instruments, Grove City, PA, USA) and a Rotator (PINE Instruments). A glassy carbon electrode (0.197 cm^2^) was used as a rotating working electrode and the rotating disk voltammograms were recorded at the scan rate of 10 mV/s by changing the rotational speed of each WE. The recorded working potentials vs. Ag|AgCl (3M KCl) were converted into the reversible hydrogen electrode (RHE) according to the equation *E*(_RHE)_ = *E*_(Ag|AgCl_) + 0.059 pH + 0.210 V, where pH (0.1M KCl) = 5.8.

To drop-cast the polymer and metallopolymer on the rotating glassy carbon electrode, the solutions were prepared by dissolving 10 mg of each material in 10 mL ethanol in the sonicator at 40 °C (sealed vial) for 1 h. After cooling down to room temperature (*RT*), the material was then drop-casted on the electrode at 200 rpm (rotations per minute) using 10 μL Eppendorf pipette and dried under ambient conditions at 200 rpm for 15 min. The prepared electrodes were then placed in the oven at 60 °C for another 20 min to achieve complete dryness of the films. The drop-casting was repeated until each electrode was fully covered by a visible film. For the polymer-only electrode, a 3 × 10 mL solution (ink) was drop-casted resulting in a mass loading of 153 µg/cm^2^ in total. To obtain the same surface area with a fully covered Cu MP, 5–6 × 10 µL solutions were drop-casted and dried under the same conditions. Since the metallopolymer precipitated in the stock-solution (10 mL vial) over time, the total mass loading was calculated to be less than 255–306 µg/cm^2^ Cu MP.

### 2.2. Polymer Synthesis

Thiophene (3 mL, 2.85 g, 34 mmol), 2,5-dihydroxybenzaldehyde/gentisaldehyde (4.7 g, 34 mmol), and 2 mL sulfuric acid (98%) were dissolved in 80 mL 1,4-dioxane and refluxed at 85 °C for 2 days. The reaction mixture became gradually dark red and resulted in a black solution after the reaction was completed. The crude solution was poured into 300 mL ice-cooled deionized water and the precipitate was washed two times with cold H_2_O. After removing all volatiles until dryness was reached on the rotary evaporator, the crude product was purified via Soxhlet extraction by washing off oligomers, unreacted reagents, and side products in acetone at 60 °C. After collecting the residuals from the cellulose thimble and drying on the vacuum pump for 5 h, 3 g of black solid were obtained. The poly-hydroquinoinyl-(thiophene) methine, PThPHQ, reacted gradually with molecular oxygen resulting in quinone derivatives. The reduced form of PThPHQ was well soluble in methanol, ethanol, and isopropanol, whereas the solubility of the oxidized form decreased in the above-mentioned alcohols.

Note: To avoid oxidation of the polymer, permanent storage under protecting gas is required.

1H NMR (300 MHz, TMS, dmso-d6): δ (ppm) = 2.8–5.5 (thiophene H and OH) and 6.5–7.8 (quinone/hydroquinone H). Due to strong overlapping, chemical shifts were not assigned.

### 2.3. Metallopolymer Synthesis

1 g (1.93 mmol polymer repetition unit) of the polymer was dissolved with 1.6 g Cu(BF_4_)_2_ 2H_2_O (5.8 mmol, 3.01 equiv. to polymer repetition unit) in 30 mL ethanol and refluxed for 3 h. After precipitation of a dark brown solid, the solvent was removed on the rotary evaporator and the crude product was washed three times with both ethanol and acetone. After removal of all volatiles until dryness, 2.2 g of dark brown solid was yielded.

## 3. Results and Discussion

For the synthesis of the polymer, the authors followed a simple one-pot synthesis procedure presented by Jenekhe et al., using 1,4-hydroquinone 2-carboxaldehyde and thiophene as reagents [50]. The black polymer obtained was purified by extracting unreacted starting materials and oligomer residuals with dichloromethane (CH_2_Cl_2_) and characterized by ^1^H NMR, UV/vis and XPS as well as SEM, respectively. The Cu(II) metallopolymer (Cu MP) was obtained as a black brownish powder by refluxing the polymer with Cu(BF_4_)_2_ in ethanol for 3 h and evaporating all volatiles until dryness.

**Figure 1:**
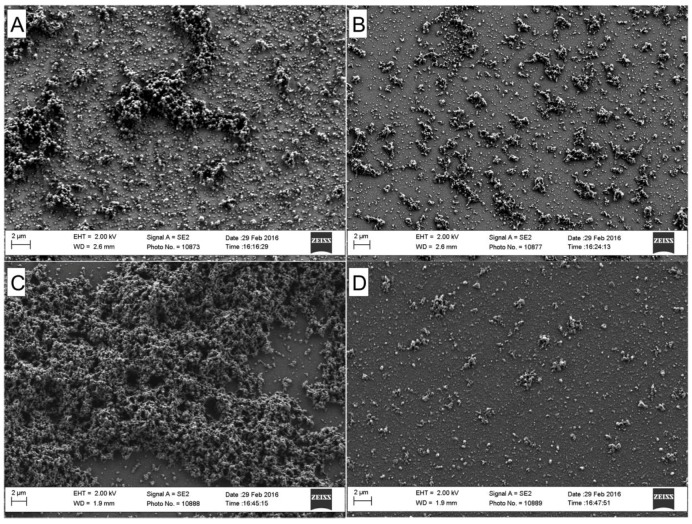
Scanning electron microscopy (SEM) images of the (**A**) drop-casted and (**B**) spin-coated PThPHQ polymer and its copper–metallopolymer, [(PThPHQ)Cu](BF_4_)_2_, (**C**) drop-casted and (**D**) spin-coated, respectively.

Figure 1A–D shows the images of the polymer and the Cu MP taken by SEM on drop-casted and spin-coated samples. The surface morphology of the drop-casted polymer samples exhibited more globular agglomerates (see Figure 1A), whereas the Cu MP resulted in highly porous structures (see Figure 1C), indicating a change in the chemical composition. Due to the fast evaporation of the solvents during spin coating at 1000 rpm, both samples showed similar structures (see Figure 1B,D). More interestingly, the Cu MP seemed to cover the whole surface area (see Figure 1D), whereas the metal-free polymer showed more islands of agglomerates (see Figure 1C).

**Figure 2:**
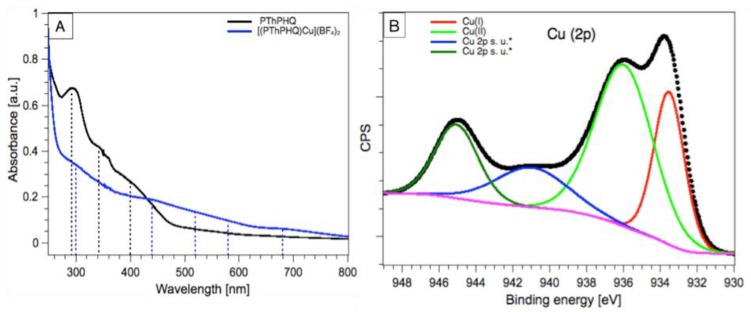
UV-Vis spectra of (**A**) PThPHQ and its metallopolymer and the 2p binding energies; of the (**B**) Cu metallopolymer; * s. u.—shake-up peaks (Cu 2p).

The UV/vis spectroscopy investigation of the polymer showed three distinct absorption bands of the polymer at 295, 352, and 400 nm that disappeared after complexation with Cu(II) (see Figure 2A). The resulting black brownish metallopolymer showed three broad absorbance bands at 300, 452, and 680 nm, indicating successful complexation with the metal centers. The recorded absorbance bands of the Cu MP point to copper–phenoxyl species, where copper is present in the oxidation state +I [58,59,60].

In fact, the deconvoluted survey spectrum of the metallopolymer’s XPS data resulted in a surprisingly high content (33%) of copper being present in the oxidation state +I (see Figure 2B). Most likely, the reduction of copper was triggered by the redox behavior of the quinone/hydroquinone units, which formed together with the central thiophene units’ coordination spheres around the Cu(II) centers.

**Figure 3:**
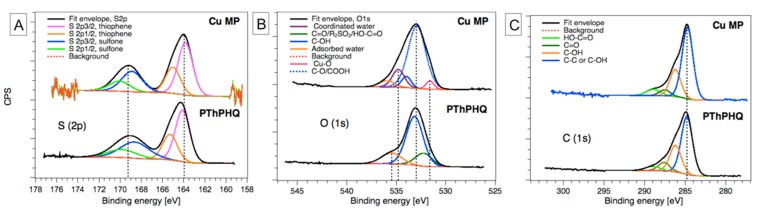
(**A**) S2p, (**B**) O1s, and (**C**) C1s core-level spectra of the analyzed Cu MP (top) compared with the polymer PThPHQ (bottom).

An in-depth analysis of the binding energies of the donors O and S in the Cu MP and polymer samples showed significant changes of the core-level spectra after complexation, where the Cu–O and coordinated water were fitted to the O(1s) envelope (see Figure 3B) in the metallopolymer sample. In contrast to the polymer, the quinonoidal C=O was not fitted to the Cu MP data at all. Instead, the hydroquinone units appeared in the O1(s) core-level spectra in their reduced form C–O and C–OH, respectively. The deconvolution of the C(1s) core-level spectra of both samples did not show any significant changes in functional groups except for the slight decrease of the binding energies (see Figure 3C). However, the S (2p) core-level spectra showed two distinct binding energies centered at 164 and 169 eV in the ratio of 2/1 for the pure polymer and its Cu MP (see Figure 3A). Latter binding energy is assigned to sulfone derivatives (RR’SO_2_) [61,62], indicating intramolecular self-oxidation of the polymer with molecular oxygen. It is likely that reactive oxygen species were generated at the pendant quinone/hydroquinone units and immediately being quenched by the central thiophene rings resulting in their oxidation. These experimental findings prompted the authors to investigate the new polymer and its inorganic hybrid further toward the electrocatalytic reduction of molecular oxygen.

**Figure 4:**
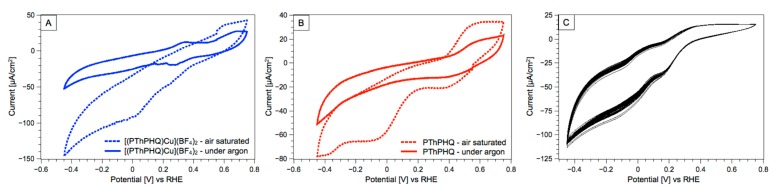
Cyclic voltammograms of the (**A**) copper metallopolymer and the polymer; (**B**) in an air- as well as argon-saturated 0.1M KCl solution (scan rate 100 mV/s, −0.45 to +0.75 V vs. RHE; (**C**) long-term stability tests of the Cu MP in the air-saturated solution and drop-casted on a gold substrate (scan rate 10 mV/s).

For preliminary ORR investigations, the polymer and its inorganic Cu hybrid were each drop-casted on a glassy carbon electrode (surface area 0.167 cm^2^), and examined in an argon- and air-saturated 0.1 molar aqueous KCl solution using cyclic voltammetry (see Figure 4A,B). For both films, reduction events were recorded at negative potentials in the air-saturated solutions. The PThPHQ polymer and the Cu MP showed activities for the ORR in the potential range of −0.45 to 0.55 V (vs. RHE), indicating their activities in the electrocatalytic ORR.

The results in the argon-saturated solutions showed much lower currents in the same potential window. Additionally, the Cu MP films showed comparably higher currents under cathodic reduction resulting in an end-current of −144 μA/cm^2^. Due to the metal doping, these voltammograms displayed more redox features compared to the metal-free ones. Long-term sweepings in the air-saturated 0.1M KCl solution for over 2 days at low scan rates (10 mV/s) between 0.75 and −0.45 V (vs. RHE) showed the metallopolymer still being attached to the electrode. However, leaching effects caused by strong turbulences (i.e., bubbling air/gas into the solution) were inevitable.

**Figure 5:**
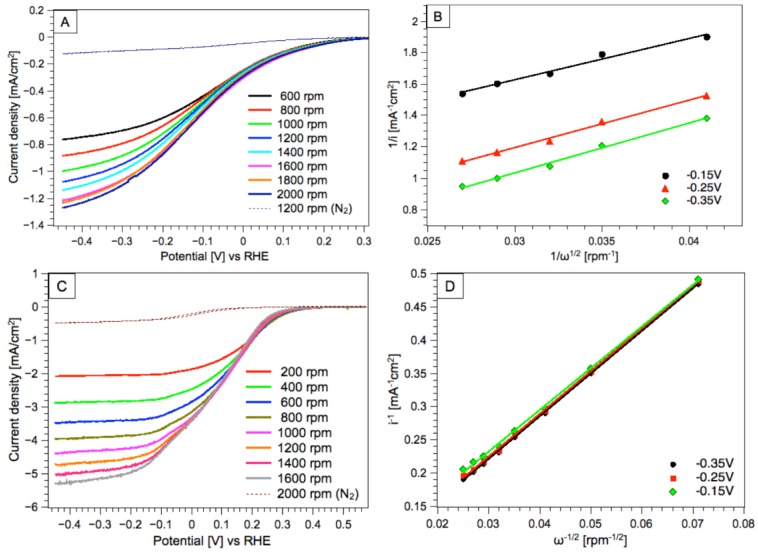
(**A**) Rotating disk voltammogram of the pure polymer PThPHQ at the rotating disk electrode (RDE) and (**B**) its obtained Koutecky–Levich plot at the potentials −0.15, −0.25, and −0.35 V vs. RHE; (**C**) rotating disk voltammogram of the copper metallopolymer [(PThPHQ)Cu](BF_4_)_2_ at the rotating disk electrode (RDE) and (**D**) its obtained Koutecky–Levich plot at the potentials −0.15, −0.25, and −0.35 vs. RHE V. The voltammograms under O_2_- (200–1600 rpm) and N_2_-saturation (2000 rpm) were obtained at 10 mV/s and under ambient conditions. For better clarity, only half cycles are shown.

The catalytic performance of the conducting polymer PThPHQ and of its metal hybrid, [(PThPHQ)Cu](BF_4_)_2_, was assessed using a rotating disk electrode (RDE). Figure 5A–D shows the rotating disk voltammograms of the films in O_2_-saturated 0.1M KCl solutions and their corresponding Koutecky–Levich plots obtained from the RDE results. Note that the drop-casted Cu MP came off the rotating glassy carbon electrode at high rotational velocities when the performance tests were conducted under alkaline (0.1M KOH) and acidic (0.1M H_2_SO_4_) conditions. Mild pH conditions appeared as more suitable for the Cu MP film to remain on the rotating electrode surface.

As shown in Figure 5A,C, both electrode materials showed increased reduction currents under O_2_ saturation and as a function of the rotational velocity, indicating their activities toward the oxygen reduction. In comparison, slight negative currents were obtained under N_2_ gas (at 2000 rpm) on both films, which was most likely caused by the formation of hydroquinone radical anions. At the Cu MP electrode, the hydroquinone radical anions appeared under O_2_ conditions as first reduction events at increased rotational velocity, resulting in an intermediate plateau at approximately 0.05 V vs. RHE. This phenomenon clearly indicated the naturally much faster intramolecular reduction of the polymer (accommodation of hydroquinone radical anions) before the intermolecular charge transfer between the metal and molecular O_2_ took place. The linearity of the plots (reciprocal current density, 1/i, versus the reciprocal square root of the rotation rate, 1/ω^1/2^) and their similar slopes confirmed the first-order kinetics with respect to the O_2_ concentration in the potential range of −0.15 to −0.35 V (see Figure 5B,D). The kinetic current densities for [(PThPHQ)Cu](BF_4_)_2_ were calculated from the intercept of the Koutecky–Levich plots to be 22.62–34.60 mA/cm^2^. For PThPHQ, the kinetic current densities of the polymer were accordingly 1.19–12.76 mA/cm^2^. Knowing that the potential ranges of −150 mV to −350 mV vs. RHE appeared as high overpotentials compared to most reported ORR catalysts systems, the kinetic current density still remained as the most important feature of an ORR catalyst at a given potential and in the absence of mass transfer limitations. Hence, the kinetic current densities of the study’s Cu MP catalyst obtained at room temperature were surprisingly high and indicated superior activities over platinum-group metals (PGMs), such as Vulcan-XC-72-supported Pd, Rh, and PdxRhy [63] and carbon-supported Pt and PtRu catalysts, respectively [64].

The number of electrons involved in the reduction reaction of each oxygen molecule was calculated from the Koutecky-Levich Equation (1),

(1) 1j=1jL+1jK=1Bω+1jK=1(−0.62 nFC0 D023ν−16)ω+1jK

where *j* is the measured current density, *j_K_* and *j_L_* are the kinetic and diffusion limiting current densities, ω is the angular velocity of the disk (*ω* = 2π*N*, is the linear rotation speed), *n* is the overall number of electrons transferred in the oxygen reduction reaction, *F* (96,485 C/mol) is the Faraday constant, *C*_0_ is the solubility (0.242 mM), and *D*_0_ is the diffusion coefficient (2.10 × 10^−5^ cm^2^/s) of O_2_ in the 0.1M KCl solution [65]; *υ* is the kinematic viscosity of the medium (0.1M KCl solution saturated with O_2_) and was measured to be 0.98 mm^2^/s.

**Figure 6:**
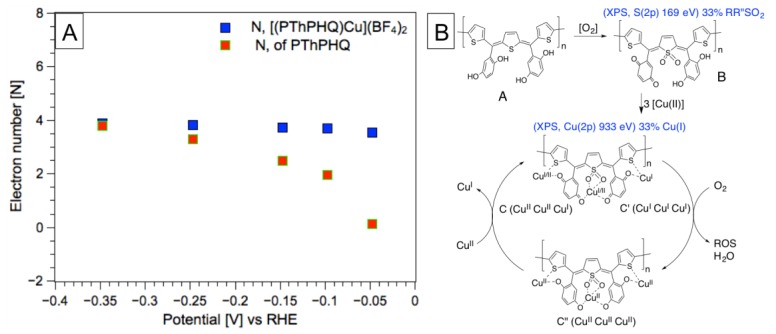
(**A**) Number of electrons (N) for the polymer and the copper metallopolymer calculated at different reduction potentials and the sketched mechanism for the electrocatalytic oxygen reduction reaction (ORR) at the Cu MP–liquid interface proposed in this study: (**B**) (i) self-oxidation B of the central thiophene units in the as-prepared PThPHQ polymer A showing a thiophene to sulfone ratio of 2/1 in the XPS; (ii) upon addition of Cu(II) to the polymer the remaining hydroquinone units undergo full oxidation to quinone (C) and reduces 1/3 of Cu(II) to Cu(I) explaining 33% of Cu(I) species in the XPS survey spectrum; (iii) in the first cycle of the electrocatalytic ORR, molecular oxygen and all quinone units are reduced to ROS, H_2_O and hydroquinone, respectively (C″); (iv) in the subsequent step, all Cu(II) centers are reduced to Cu(I) in the copper-catalyzed hydroquinone oxidation [67] (C2023), which then become available for the next cycling of the oxygen reduction (C″).

Figure 6A shows the average number of electrons in the ORR for the Cu MP and the polymer. The Cu MP catalyst accelerates a nearly four-electron reduction in the full potential range between −0.05 and −0.35 V vs. RHE. Thus, a direct reduction of oxygen into water was obtained. In comparison, the polymer PThPHQ showed a smooth transition from a two-electron reduction at −0.1 to −0.15 to a four-electron reduction at the end potentials (−0.25 to −0.35 V) suggesting that hydrogen peroxide was being formed as an intermediate product (see Figure 6A, red). Since hydroquinone and its derivatives are known materials for the electrocatalytic hydrogen peroxide production [66], the authors propose a two-electron reduction path of molecular oxygen at hydroquinone-metal units into hydrogen peroxide (H_2_O_2_) intermediates with a subsequent oxidation of Cu(I) to Cu(II) [67], resulting in the formation of water as the end product (see Figure 6B). In an operating proton exchange membrane (PEM) fuel cell, H_2_O_2_ that is formed as a side-product and the subsequent decomposition to reactive oxygen species are considered the major source for the membrane degradation [68,69,70]. Hence, ORR catalysts with a direct four-electron reduction toward molecular oxygen are highly regarded. They not only exhibit high power densities, but also extend the performance and lifetime the fuel cells.

In the table below, the performance data of the Cu MP electrocatalyst are compared to some selected benchmark systems. Overall, the metallopolymer electrode required higher overpotentials to catalyze molecular oxygen in the four-electron reduction process. However, its kinetic current density was almost twice as high as the current obtained on benchmark catalysts such as carbon-supported Pt (Pt/C) and nitrogen-doped porous carbon nanosheets (NPCN). To the best of the authors’ knowledge, it is the highest reported performance parameter so far. The reduction potential of the Cu MP (vs. RHE) is still comparable to the potentials recorded on transition-metals carbide supported on nitrogen-doped graphene (FeMo-NG). Metal-free electrocatalysts such as GC-N800 and g-C3N4@C are also reported as highly efficient electrocatalysts toward ORR at slightly lower overpotentials (see Table 1), but their kinetic current per surface area are still far below the activity of Pt/C and the Cu MP catalyst in this study’s work.

## 4. Conclusions

This work demonstrated the potential use of metallopolymers as catalysts for the molecular oxygen to water reduction at moderate potentials. Rotating disk measurements showed a direct four-electron reduction process of the metallopolymer at −150 to −350 mV vs. RHE, where the polymer contained single copper sites as catalytic centers. Long-term stability experiments in 0.1M KCl for up to two days were monitored without significant loss of activity. The authors hypothesized that the stability was caused by the combination of hard (oxygen) and soft (sulfur) donors during the electrochemical redox catalysis. Surface analysis of the metallopolymer prior to electrocatalytic investigations by XPS revealed a third of the copper being reduced to Cu(I), which was driven by the redox nature of the quinone-hydroquinone couple. The high redox nature of the quinone-hydroquinone was again highlighted by the autocatalytic self-oxidation at the thiophene backbone. The authors believe that the high kinetic current densities of the Cu MP electrocatalysts are a consequence of the interplay between the Cu(II/I) redox catalysis and the redox nature of the quinone/hydroquinone units of the conducting polymer, which is itself an ORR catalyst.

## Figures and Tables

**Table 1:** Performance data (onset reduction potential *E*_red,ons_, number of electrons N, and kinetic current density *I*_red,ons_ at the selected onset reduction potential) of the Cu MP electrode compared to some selected electrodes from the literature.

Catalyst	Electrolyte	*E*_red,ons_ (V vs. RHE) ^(a)^	*I*_red,ons_ (mA/cm^2^)	*N*	Lit.
Fe3C/Fe@G-800	0.1M KOH	0.91	n.r. ^(^^b)^	3.71	[71]
NPCN	0.1M KOH	0.72	13.57	3.70	[72]
Pt/C	0.1M KOH	0.72–0.8	9.28–14.00	4.00	[64,72,73]
GC-N800	0.1M KOH	0.58	7.30	4.00	[74]
g-CN3@C	0.1M KOH	0.37	11.30	3.90	[75]
FeMo-NG	0.1M KOH	0.17	3.50	3.90	[76]
PdRh/C, Rh/C	0.5M H_2_SO_4_	0.70 ^(c)^	0.89–5.20	4.00	[63]
Cu MP	0.1M KCl	−0.15	22.62–30.78	3.75–3.90	This work

**^(a^**^)^—The reduction potential *E*_red_ vs. RHE was calculated from the Ag|AgCl according *E*_(vs. RHE)_ = *E*_(vs. Ag|AgCl)_ + 0.059 pH + *E*^0^_(Ag|AgCl)_; ^(b)^—not reported; ^(c)^—referred to the standard calomel electrode (vs. SCE).

## References

[1] Wang J., Chen C.S., Zhang Y. (2018). Hexaazatrinaphthylene-Based Porous Organic Polymers as Organic Cathode Materials for Lithium-Ion Batteries. ACS Sustain. Chem. Eng..

[2] Liu H., Wang J.-G., Hua W., Wang J., Nan D., Wei C. (2018). Scale-up production of high-tap-density carbon/MnOx/carbon nanotube microcomposites for Li-ion batteries with ultrahigh volumetric capacity. Chem. Eng. J..

[3] Wang J.-G., Sun H., Liu H., Jin D., Liu X., Li X., Kang F. (2018). Triaxial Nanocables of Conducting Polypyrrole@SnS2@Carbon Nanofiber Enabling Significantly Enhanced Li-Ion Storage. ACS Appl. Mater. Interfaces.

[4] Wang X., Chen S., Li D., Sun S., Peng Z., Komarneni S., Yang D. (2018). Direct Interfacial Growth of MnO_2_ Nanostructure on Hierarchically Porous Carbon for High-Performance Asymmetric Supercapacitors. ACS Sustain. Chem. Eng..

[5] Wang J.-G., Liu H., Liu H., Hua W., Shao M. (2018). Interfacial Constructing Flexible V2O5 @Polypyrrole Core–Shell Nanowire Membrane with Superior Supercapacitive Performance. ACS Appl. Mater. Interfaces.

[6] Wang J.-G., Liu H., Sun H., Hua W., Wang H., Liu X., Wei B. (2018). One-pot synthesis of nitrogen-doped ordered mesoporous carbon spheres for high-rate and long-cycle life supercapacitors. Carbon.

[7] Sahoo S., Shim J.-J. (2017). Facile Synthesis of Three-Dimensional Ternary ZnCo_2_O_4_/Reduced Graphene Oxide/NiO Composite Film on Nickel Foam for Next Generation Supercapacitor Electrodes. ACS Sustain. Chem. Eng..

[8] Li G., Shrotriya V., Huang J., Yao Y., Moriarty T., Emery K., Yang Y. (2005). High-efficiency solution processable polymer photovoltaic cells by self-organization of polymer blends. Nat. Mater..

[9] Garg R., Elmas S., Nann T., Andersson M.R. (2017). Deposition Methods of Graphene as Electrode Material for Organic Solar Cells. Adv. Energy Mater..

[10] Lin X.-X., Wang A.-J., Fang K.-M., Yuan J., Feng J.-J. (2017). One-Pot Seedless Aqueous Synthesis of Reduced Graphene Oxide (rGO)-Supported Core–Shell Pt@Pd Nanoflowers as Advanced Catalysts for Oxygen Reduction and Hydrogen Evolution. ACS Sustain. Chem. Eng..

[11] Hsu P.-Y., Hu T.-Y., Kumar S., Chang C.-H., Wu K., Tung K.-L., Lue S. (2018). Highly Zeolite-Loaded Polyvinyl Alcohol Composite Membranes for Alkaline Fuel-Cell Electrolytes. Polymers.

[12] Brian C.H. (2001). Steele Angelika Heinzel Materials for fuel cell technologies. Nat. Mater..

[13] Winter M., Brodd R.J. (2004). What Are Batteries, Fuel Cells, and Supercapacitors?. Chem. Rev..

[14] Stambouli A.B., Traversa E. (2002). Solid oxide fuel cells (SOFCs): A review of an environmentally clean and efficient source of energy. Renew. Sustain. Energy Rev..

[15] Qu L., Liu Y., Baek J.-B., Dai L. (2010). Nitrogen-Doped Graphene as Efficient Metal-Free Electrocatalyst for Oxygen Reduction in Fuel Cells. ACS Nano.

[16] Yu E. (2004). Development of direct methanol alkaline fuel cells using anion exchange membranes. J. Power Sources.

[17] Borup R., Meyers J., Pivovar B., Kim Y.S., Mukundan R., Garland N., Myers D., Wilson M., Garzon F., Wood D. (2007). Scientific Aspects of Polymer Electrolyte Fuel Cell Durability and Degradation. Chem. Rev..

[18] Atkinson A., Barnett S., Gorte R.J., Irvine J.T.S., McEvoy A.J., Mogensen M., Singhal S.C., Vohs J. (2004). Advanced anodes for high-temperature fuel cells. Nat. Mater..

[19] Zhang J., Sasaki K., Sutter E., Adzic R.R. (2007). Stabilization of Platinum Oxygen-Reduction Electrocatalysts Using Gold Clusters. Science.

[20] Iijima Y., Kondo T., Takahashi Y., Bando Y., Todoroki N., Wadayama T. (2013). Oxygen Reduction Reaction Activities for Pt/Au(hkl) Bimetallic Surfaces Prepared by Molecular Beam Epitaxy. J. Electrochem. Soc..

[21] Chen G., Zhao Y., Fu G., Duchesne P.N., Gu L., Zheng Y., Weng X., Chen M., Zhang P., Pao C.-W. (2014). Interfacial Effects in Iron-Nickel Hydroxide-Platinum Nanoparticles Enhance Catalytic Oxidation. Science.

[22] Wang C., Markovic N.M., Stamenkovic V.R. (2012). Advanced Platinum Alloy Electrocatalysts for the Oxygen Reduction Reaction. ACS Catal..

[23] Gong K., Yu P., Su L., Xiong S., Mao L. (2007). Polymer-Assisted Synthesis of Manganese Dioxide/Carbon Nanotube Nanocomposite with Excellent Electrocatalytic Activity toward Reduction of Oxygen. J. Phys. Chem. C.

[24] Donne S.W. (1997). Redox Processes at the Manganese Dioxide Electrode. J. Electrochem. Soc..

[25] Gong K., Du F., Xia Z., Durstock M., Dai L. (2009). Nitrogen-Doped Carbon Nanotube Arrays with High Electrocatalytic Activity for Oxygen Reduction. Science.

[26] Kongkanand A., Kuwabata S., Girishkumar G., Kamat P. (2006). Single-Wall Carbon Nanotubes Supported Platinum Nanoparticles with Improved Electrocatalytic Activity for Oxygen Reduction Reaction. Langmuir.

[27] Guangli C., Brinda B.L., Ellen R.F., Charles L. (1998). Martin Carbon nanotubule membranes for electrochemical energy storage and production. Nature.

[28] Coleman E.J., Chowdhury M.H., Co A.C. (2015). Insights into the Oxygen Reduction Reaction Activity of Pt/C and PtCu/C Catalysts. ACS Catal..

[29] Dai L., Xue Y., Qu L., Choi H.-J., Baek J.-B. (2015). Metal-Free Catalysts for Oxygen Reduction Reaction. Chem. Rev..

[30] Chai G.-L., Boero M., Hou Z., Terakura K., Cheng W. (2017). Indirect Four-Electron Oxygen Reduction Reaction on Carbon Materials Catalysts in Acidic Solutions. ACS Catal..

[31] Gong X., Liu S., Ouyang C., Strasser P., Yang R. (2015). Nitrogen- and Phosphorus-Doped Biocarbon with Enhanced Electrocatalytic Activity for Oxygen Reduction. ACS Catal..

[32] Halime Z., Kotani H., Li Y., Fukuzumi S., Karlin K.D. (2011). Homogeneous catalytic O2 reduction to water by a cytochrome c oxidase model with trapping of intermediates and mechanistic insights. Proc. Natl. Acad. Sci. USA.

[33] Collman J.P., Devaraj N.K., Decreau R.A., Yang Y., Yan Y.-L., Ebina W., Eberspacher T.A., Chidsey C.E.D. (2007). A Cytochrome c Oxidase Model Catalyzes Oxygen to Water Reduction Under Rate-Limiting Electron Flux. Science.

[34] Parimi N.S., Umasankar Y., Atanassov P., Ramasamy R.P. (2012). Kinetic and Mechanistic Parameters of Laccase Catalyzed Direct Electrochemical Oxygen Reduction Reaction. ACS Catal..

[35] Winther-Jensen B., Winther-Jensen O., Forsyth M., MacFarlane D.R. (2008). High Rates of Oxygen Reduction over a Vapor Phase-Polymerized PEDOT Electrode. Science.

[36] Barsukov V., Chivikov S. (1996). The “capacitor” concept of the current-producing process mechanism in polyaniline-type conducting polymers. Electrochim. Acta.

[37] Khomenko V.G., Barsukov V.Z., Katashinskii A.S. (2005). The catalytic activity of conducting polymers toward oxygen reduction. Electrochim. Acta.

[38] Tylus U., Jia Q., Strickland K., Ramaswamy N., Serov A., Atanassov P., Mukerjee S. (2014). Elucidating Oxygen Reduction Active Sites in Pyrolyzed Metal–Nitrogen Coordinated Non-Precious-Metal Electrocatalyst Systems. J. Phys. Chem. C.

[39] Bouwkamp-Wijnoltz A.L., Visscher W., van Veen J.A.R., Boellaard E., van der Kraan A.M., Tang S.C. (2002). On Active-Site Heterogeneity in Pyrolyzed Carbon-Supported Iron Porphyrin Catalysts for the Electrochemical Reduction of Oxygen: An In Situ Mössbauer Study. J. Phys. Chem. B.

[40] Koslowski U.I., Abs-Wurmbach I., Fiechter S., Bogdanoff P. (2008). Nature of the Catalytic Centers of Porphyrin-Based Electrocatalysts for the ORR: A Correlation of Kinetic Current Density with the Site Density of Fe−N 4 Centers. J. Phys. Chem. C.

[41] Li Y., Zhou W., Wang H., Xie L., Liang Y., Wei F., Idrobo J.-C., Pennycook S.J., Dai H. (2012). An oxygen reduction electrocatalyst based on carbon nanotube–graphene complexes. Nat. Nanotechnol..

[42] Genorio B., Strmcnik D., Subbaraman R., Tripkovic D., Karapetrov G., Stamenkovic V.R., Pejovnik S., Marković N.M. (2010). Selective catalysts for the hydrogen oxidation and oxygen reduction reactions by patterning of platinum with calix[4]arene molecules. Nat. Mater..

[43] Zheng Q., Cheng X., Jao T.-C., Weng F.-B., Su A., Chiang Y.-C. (2012). Degradation analyses of Ru85Se15 catalyst layer in proton exchange membrane fuel cells. J. Power Sources.

[44] Wu J., Yuan X.Z., Martin J.J., Wang H., Zhang J., Shen J., Wu S., Merida W. (2008). A review of PEM fuel cell durability: Degradation mechanisms and mitigation strategies. J. Power Sources.

[45] Kingsborough R.P., Swager T.M. (2000). Electrocatalytic Conducting Polymers: Oxygen Reduction by a Polythiophene−Cobalt Salen Hybrid. Chem. Mater..

[46] Elmas S., Beelders W., Bradley S.J., Kroon R., Laufersky G., Andersson M., Nann T. (2017). Platinum Terpyridine Metallopolymer Electrode as Cost-Effective Replacement for Bulk Platinum Catalysts in Oxygen Reduction Reaction and Hydrogen Evolution Reaction. ACS Sustain. Chem. Eng..

[47] Elmas S., Beelders W., Nash J., Macdonald T.J., Jasieniak M., Griesser H.J., Nann T. (2016). Photo-doping of plasma-deposited polyaniline (PAni). RSC Adv..

[48] Heeger A.J. (2001). Semiconducting and Metallic Polymers: The Fourth Generation of Polymeric Materials (Nobel Lecture). Angew. Chem. Int. Ed..

[49] Kingsborough R.P., Swager T.M. (1998). Electroactivity Enhancement by Redox Matching in Cobalt Salen-Based Conducting Polymers. Adv. Mater..

[50] Chen W.-C., Jenekhe S.A. (1995). Small-Bandgap Conducting Polymers Based on Conjugated Poly(heteroarylene methines). 2. Synthesis, Structure, and Properties. Macromolecules.

[51] Zhang Q., Feng J., Liu K., Zhu D., Yang M., Ye H., Liu X. (2006). Synthesis and characterization of novel low band gap polymers: Poly(heteroarylene methines). Synth. Met..

[52] Yi W., Feng W., Cao M., Wu H. (2004). Synthesis of third-order non-linear optical polymers based on conjugated poly(heteroarylene methines). Polym. Adv. Technol..

[53] de Melo B.A.G., Motta F.L., Santana M.H.A. (2016). Humic acids: Structural properties and multiple functionalities for novel technological developments. Mater. Sci. Eng. C.

[54] Larsson M., Yousefi A., Elmas S., Lindén J.B., Nann T., Nydén M. (2017). Electroactive Polyhydroquinone Coatings for Marine Fouling Prevention—A Rejected Dynamic pH Hypothesis and a Deceiving Artifact in Electrochemical Antifouling Testing. ACS Omega.

[55] Vonlanthen D., Lazarev P., See K.A., Wudl F., Heeger A.J. (2014). A Stable Polyaniline-Benzoquinone-Hydroquinone Supercapacitor. Adv. Mater..

[56] Klein A., Elmas S., Butsch K. (2009). Oxido Pincer Ligands–Exploring the Coordination Chemistry of Bis(hydroxymethyl)pyridine Ligands for the Late Transition Metals. Eur. J. Inorg. Chem..

[57] Klein A., Butsch K., Elmas S., Biewer C., Heift D., Nitsche S., Schlipf I., Bertagnolli H. (2012). Oxido-pincer complexes of copper(II)–An EXAFS and EPR study of mono- and binuclear [(pydotH2)CuCl2]n (n = 1 or 2). Polyhedron.

[58] Zhang L., Yue S., Li B., Fan D. (2012). A series of [Cu(N–N)(P–P)]BF4 complexes: Luminescence quenching caused by electron-configuration transformation in excited state. Inorg. Chim. Acta.

[59] Megiatto J.D., Schuster D.I. (2011). Alternative Demetalation Method for Cu(I)-Phenanthroline-Based Catenanes and Rotaxanes. Org. Lett..

[60] Klein A., Butsch K., Neudörfl J. (2010). Electron transfer studies on Cu(II) complexes bearing phenoxy-pincer ligands. Inorg. Chim. Acta.

[61] Wavhal D.S., Fisher E.R. (2002). Hydrophilic modification of polyethersulfone membranes by low temperature plasma-induced graft polymerization. J. Membr. Sci..

[62] Wang H., Cheng F., Li M., Peng W., Qu J. (2015). Reactivity and Kinetics of Vinyl Sulfone-Functionalized Self-Assembled Monolayers for Bioactive Ligand Immobilization. Langmuir.

[63] Tzorbatzoglou F., Brouzgou A., Tsiakaras P. (2015). Electrocatalytic activity of Vulcan-XC-72 supported Pd, Rh and PdxRhy toward HOR and ORR. Appl. Catal. B Environ..

[64] Jiang L., Hsu A., Chu D., Chen R. (2009). Oxygen reduction on carbon supported Pt and PtRu catalysts in alkaline solutions. J. Electroanal. Chem..

[65] Ikeuchi H., Hayafuji M., Aketagawa Y., Taki J., Sato G.P. (1995). Diffusion coefficients of dioxygen in aqueous electrolyte solutions. J. Electroanal. Chem..

[66] Tammeveski K., Kontturi K., Nichols R.J., Potter R.J., Schiffrin D.J. (2001). Surface redox catalysis for O_2_ reduction on quinone-modified glassy carbon electrodes. J. Electroanal. Chem..

[67] Yuan X., Pham A.N., Miller C.J., Waite T.D. (2013). Copper-Catalyzed Hydroquinone Oxidation and Associated Redox Cycling of Copper under Conditions Typical of Natural Saline Waters. Environ. Sci. Technol..

[68] Gubler L., Dockheer S.M., Koppenol W.H. (2011). Radical (HO●, H● and HOO●) Formation and Ionomer Degradation in Polymer Electrolyte Fuel Cells. J. Electrochem. Soc..

[69] Chen C., Fuller T.F. (2009). XPS Analysis of Polymer Membrane Degradation in PEMFCs. J. Electrochem. Soc..

[70] Shah A.A., Ralph T.R., Walsh F.C. (2009). Modeling and Simulation of the Degradation of Perfluorinated Ion-Exchange Membranes in PEM Fuel Cells. J. Electrochem. Soc..

[71] Song A., Cao L., Yang W., Li Y., Qin X., Shao G. (2018). Uniform Multilayer Graphene-Coated Iron and Iron-Carbide as Oxygen Reduction Catalyst. ACS Sustain. Chem. Eng..

[72] Pan F., Cao Z., Zhao Q., Liang H., Zhang J. (2014). Nitrogen-doped porous carbon nanosheets made from biomass as highly active electrocatalyst for oxygen reduction reaction. J. Power Sources.

[73] Chen X., He F., Shen Y., Yang Y., Mei H., Liu S., Mori T., Zhang Y. (2017). Effect of Carbon Supports on Enhancing Mass Kinetic Current Density of Fe-N/C Electrocatalysts. Chem. Eur. J..

[74] Yang S., Feng X., Wang X., Müllen K. (2011). Graphene-Based Carbon Nitride Nanosheets as Efficient Metal-Free Electrocatalysts for Oxygen Reduction Reactions. Angew. Chem. Int. Ed..

[75] Zheng Y., Jiao Y., Chen J., Liu J., Liang J., Du A., Zhang W., Zhu Z., Smith S.C., Jaroniec M. (2011). Nanoporous Graphitic-C 3 N 4 @Carbon Metal-Free Electrocatalysts for Highly Efficient Oxygen Reduction. J. Am. Chem. Soc..

[76] Chen M., Liu J., Zhou W., Lin J., Shen Z. (2015). Nitrogen-doped Graphene-Supported Transition-metals Carbide Electrocatalysts for Oxygen Reduction Reaction. Sci. Rep..

